# One-DOF Superimposed Rigid Origami with Multiple States

**DOI:** 10.1038/srep36883

**Published:** 2016-11-10

**Authors:** Xiang Liu, Joseph M. Gattas, Yan Chen

**Affiliations:** 1 School of Mechanical Engineering, Tianjin University, Tianjin 300072, China; 2 School of Civil Engineering, University of Queensland, St Lucia, QLD 4072, Australia; 3 Key Laboratory of Mechanism Theory and Equipment Design of Ministry of Education, Tianjin University, Tianjin 300072, China

## Abstract

Origami-inspired engineering design is increasingly used in the development of self-folding structures. The majority of existing self-folding structures either use a bespoke crease pattern to form a single structure, or a universal crease pattern capable of forming numerous structures with multiple folding steps. This paper presents a new approach whereby multiple distinct, rigid-foldable crease patterns are superimposed in the same sheet such that kinematic independence and 1-DOF mobility of each individual pattern is preserved. This is enabled by the cross-crease vertex, a special configuration consisting of two pairs of collinear crease lines, which is proven here by means of a kinematic analysis to contain two independent 1-DOF rigid-foldable states. This enables many new origami-inspired engineering design possibilities, with two explored in depth: the compact folding of non-flat-foldable structures and sequent folding origami that can transform between multiple states without unfolding.

Origami-inspired engineering is to apply origami science and technology to the design of engineering structures or devices with remarkable performance characteristics. Applications have been developed across most engineering disciplines, with examples including space structures^1^,^2^, deployable shelters^3^, sandwich panels^4^,^5^,^6^, meta-materials^7^,^8^, medical implants^9^,^10^, and automobile components^11^,^12^. Many of these applications utilize a rigid-foldable origami pattern, which can fold without twisting or stretching of component panels and thus can be folded from rigid engineering sheet materials^13^. A substantial body of research into understanding the mathematics and kinematics of rigid-foldable origami patterns has enabled the above engineering explorations^14^,^15^. These have shown that conditions for rigid-foldability and the degrees of freedom (DOF) are determined by the geometry of the origami crease pattern, a network of folding lines that are placed in a sheet and intersect at vertices^16^. Properties such as, the flat-foldability and large areal reduction ratio are also of great importance in practice^17^. Yet, not all the crease patterns offer a compactly flat-folded configuration and even when such a configuration is achieved, the areal reduction ratio may be limited by the geometric parameters of the pattern design.

Most existing crease patterns are for a solo pattern, for example the Miura-ori (rigid with 1-DOF^18^) or the waterbomb tessellation (rigid with multi-DOF^19^). For these, the folding process and the folded shape are clearly pre-defined by the crease patterns. There are, nevertheless, some exceptions. For example, two sets of patterns have been integrated on printed circuit boards in a Latin-cross shape without intersection such that it can be folded into a pentahedron or an octahedron, which is controlled by the actuation at each folding line through software and hardware^20^,^21^. A ‘universal’ crease pattern has also been investigated that can be dynamically programmed into different shapes by folding a subset of available creases, which is a multi-DOF process with multiple folding steps. The order of the folded states has to be followed strictly in order to obtain the desired configurations in which a programmable origami is applied^22^.

In this paper, we propose a fundamentally new method for generation of multiple 1-DOF rigid-foldable configurations from pre-defined crease patterns. The method leverages the existing knowledge surrounding rigid-foldability to enable crease patterns of different 1-DOF rigid-foldable states to be embedded within a single sheet, such that kinematic-independence is preserved. Sheets can thus be created that contain multiple states with different or complementary functionality and substantial potential for application in origami-inspired engineering design.

## Results

### Superimposed rigid-foldable patterns

**Figure 1:**
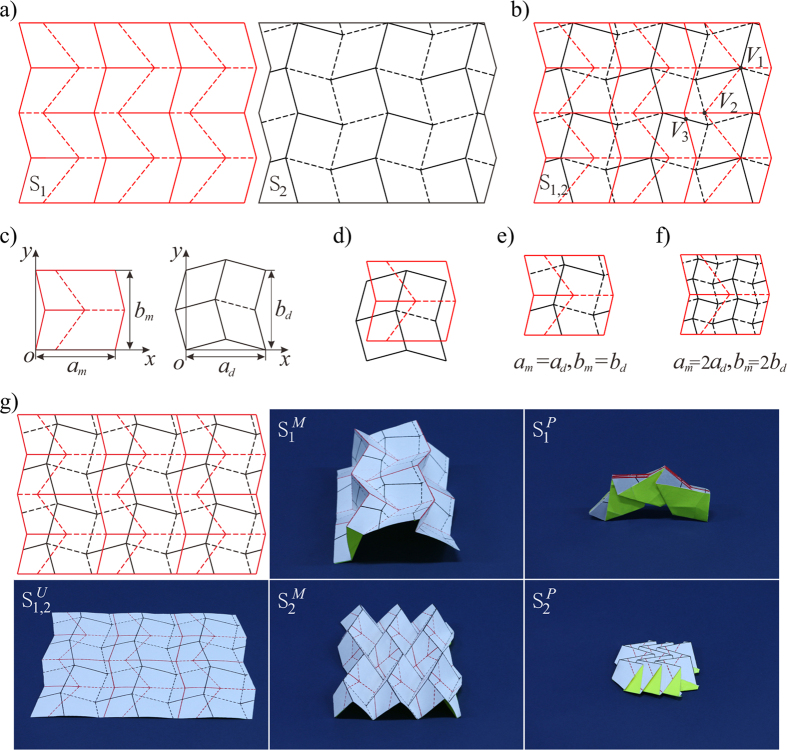
Superimposed rigid-foldable patterns. In which solid lines are mountain folds and dash lines are valley folds. (**a**) 1-DOF rigid-foldable arc-Miura pattern *S*_1_ and double corrugated pattern *S*_2_. (**b**) Patterns *S*_1_ and *S*_2_ are superimposed in the same sheet to form a pattern *S*_1,2_, in which *V*_1_ is the eight-crease combined vertex, *V*_2_ is the six-crease intersected vertex, and *V*_3_ is the four-crease cross-crease vertex. **(c**) Unit patterns of *S*_1_ and *S*_2._ (**d**) Superimposed units with dimensional shift. (**e**) Overhanging regions of *S*_2_ translated to within *S*_1_. (**f**) 2 by 2 small *S*_2_ units superimposed within a single *S*_1_ unit. (**g**) A superimposed pattern *S*_1,2_ with only additional cross-crease vertices and its prototype with two independent 1-DOF rigid-foldable states, in which superscripts U, M, D and P represents the fully unfolded, intermediate folding, deployed and fully packed states, respectively.

Shown in Fig. 1a are two origami patterns. The red pattern, *S*_1_, forms a single-curved surface and the black one, *S*_2_, forms a planar surface, both with 1-DOF rigid-foldability. *S*_1_ and *S*_2_ crease patterns are superimposed on top of each other within the same sheet to form the crease pattern of *S*_1,2_, see Fig. 1b. In order to make the folding movement of patterns *S*_1_ and *S*_2_ independent, the superimposition cannot be carried out arbitrarily. Many vertices are unchanged from the single pattern to the superimposed one. However, when *S*_1_ and *S*_2_ vertices are coincident, a *combined vertex* is formed such as the eight-crease vertex *V*_1_. According to the principles of rigid origami kinematics^23^, this vertex has a DOF of 5. Similarly, when an *S*_1_ vertex is intersected by an *S*_2_ crease line, or vice versa, an *intersected vertex* is formed such as the six-crease vertex *V*_2_, with a DOF of 3. The combined and intersected vertices thus disrupt the 1-DOF rigid-foldability of the initial patterns and thus the global rigid-foldability in the combined pattern *S*_1,2_ cannot be easily determined or controlled. The third type of new vertex *V*_3_ appears in *S*_1,2_ when two straight crease lines, one each from *S*_1_ and *S*_2_, intersect. They are here termed *cross-crease vertices*. The kinematic effect of this vertex type can be determined as follows.

In rigid origami, facets and crease lines are equivalent to rigid panels and revolute joints, respectively. As all crease lines meet at vertices, a rigid origami pattern with a single vertex is kinematically equivalent to a spherical linkage and a pattern with multiple vertices is equivalent to the assembly or network of a number of spherical linkages.

**Figure 2:**
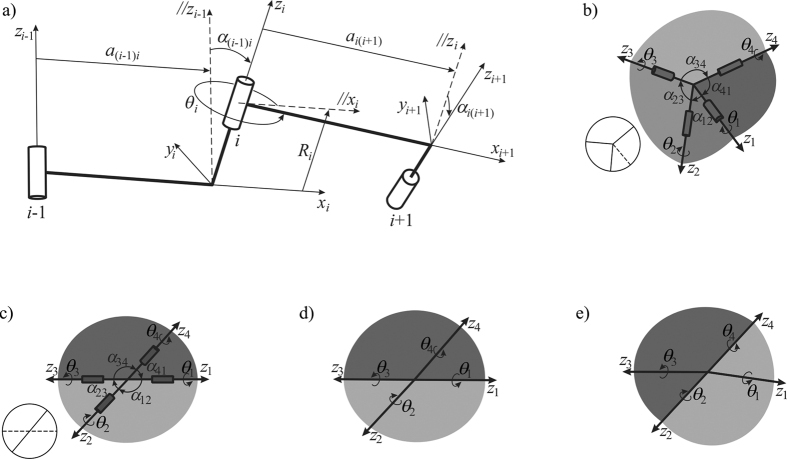
Four-crease vertex of rigid origami and corresponding spherical 4*R* linkage. (**a**) The setup of DH coordinates and kinematic parameters. (**b**) General four-crease vertex and its corresponding spherical 4*R* linkage marked with kinematic joint axes *z*_*i*_, geometric parameters *α*_(*i−*1)*i*_ and rotation variables *θ*_*i*_. (**c**) A cross-crease vertex and its corresponding kinematic model. (**d**) The folding mode of the cross-crease vertex with path *θ*_2_ = *θ*_4_ = 0, *θ*_1_ = *θ*_3_. (**e**) The folding mode of the cross-crease vertex with path 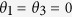, *θ*_2_ = *θ*_4_.

Therefore, the kinematic properties of rigid origami can be obtained by analyzing the corresponding spherical linkages and networks with standard kinematic theory. Here, the Denavit and Hartenberg (DH) matrix method^24^ is applied, see Fig. 2a. The axes of four revolute joints (or crease lines) are *z*_*i*_. The DH coordinates are then setup on each joint *i* along *z*-axis, where axis *x*_*i*_ is commonly normal to *z*_*i*_ and *z*_*i*−1_, and axis *y*_*i*_ is normal to *x*_*i*_ and *z*_*i*_ following the right-hand rule. Thus the kinematic geometric parameters are defined as *a*_(*i*−1)*i*_, the distance between axes *z*_*i*−1_ and *z*_*i*_, positive along *x*_*i*_ (*a*_(*i*−1)*i*_ = 0 for spherical linkages), and *α*_(*i−*1)*i*_, the angle between axes *z*_*i*−1_ and *z*_*i*_, positive along *x*_*i*_. The kinematic variable *θ*_i_ is defined as the rotation between two panels joined by the crease or revolute joint *z*_*i*_. For a closed-loop spherical linkage, shown in Fig. 2b, the necessary and sufficient mobility condition is obtained when the product of the transformation matrices equals the unit matrix, that is


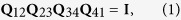


in which, **Q**_*i*(*i*+1)_ is the transformation matrix from the *i*th coordinate system on joint *i* to the (*i*+1)th coordinate system on joint (*i*+1), i.e.,


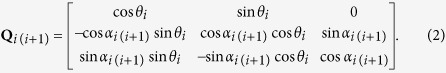


Under this framework, the rotation of the crease lines can be obtained. Yet, here our attention is on the cross-crease vertex, which can be considered kinematically as a four-crease vertex in which alternate pairs of crease lines are collinear and of the same polarity. Its corresponding linkage form is shown in Fig. 2c. The kinematic geometric parameters of this linkage are


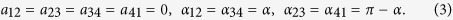


where both crease lines 1 and 3 are both either mountain folds (0 < *θ* < *π*) or valley folds (−*π* < *θ* < 0), as are crease lines 2 and 4. Substituting (3) into the closure condition (1), we can get the kinematic closure equation of the linkage in Fig. 2c as


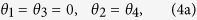


or


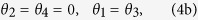


which indicates that the rigid origami pattern in the cross-crease vertex is in general 1-DOF, but in the unfolded configuration *θ*_1_ = *θ*_2_ = *θ*_3_ = *θ*_4_ = 0, a kinematic bifurcation exists with two possible moving paths, see Fig. 2d,e. However, this bifurcation behavior will not disturb the folding movement of the origami pattern and indeed can be utilized for practical benefit in superimposed origami patterns. For instance, a superimposed pattern is shown in Fig. 1g which has cross-crease vertices only, that is it has no intersected or combined vertices. The actuation of *S*_1_ will constrain all cross-crease vertices to one of two paths given in equations (4) while the other path is held inactive. Pattern *S*_1,2_ is able to independently fold between each state *S*_1_ or *S*_2_ as shown in the prototype. We can therefore conclude that if origami patterns are superimposed such that only cross-crease vertices are added in the combined pattern, kinematic independence between states and 1-DOF mobility are preserved.

To ensure all new intersections in superimposed origami patterns are cross-crease vertices, we can consider the superimposition method of the pattern units. For the arc-Miura pattern, *S*_1_ in Fig. 1a, there are 2 by 3 units, one of which is shown as red in Fig. 1c with dimensions *a*_*m*_ and *b*_*m*_. For the double corrugated pattern, *S*_2_ in Fig. 1a, there are 2 by 2 units, one of which is shown as black in Fig. 1c with dimensions *a*_*d*_ and *b*_*d*_. If units are superimposed as shown in Fig. 1d, it can easily be seen that no combined or intersected vertices are present in the superposed pattern and there are only cross crease vertices added. The overhanging regions of one state can be shifted in both directions to ensure there are no combined or intersected vertices generated when units are tessellated, shown in Fig. 1e. Then, if the dimensions of both units satisfy *a*_*m*_ = *ka*_*d*_ (or *a*_*d*_ = *ka*_*m*_) and *b*_*m*_ = *lb*_*d*_, (or *b*_*d*_ = *lb*_*m*_) where *k* and *l* are positive integers, tessellation of the superimposed units can be carried out to generate a larger origami pattern with only cross-crease vertices present. The examples *k* = *l* = 1 in Fig. 1e and *k* = *l* = 2 in Fig. 1f can both be tessellated into a larger origami pattern such as that given in Fig. 1g and SI Video 1.

### Compact folding of non-flat-foldable structures

There are a very large number of 1-DOF rigid-foldable patterns that can be combined within a single sheet using the methods described above. Rather than simply combining known 1-DOF states, it is more useful to consider how secondary patterns can be combined with a primary pattern to develop applications with extended functionality. This is discussed in the context of specific examples as follows.

**Figure 3:**
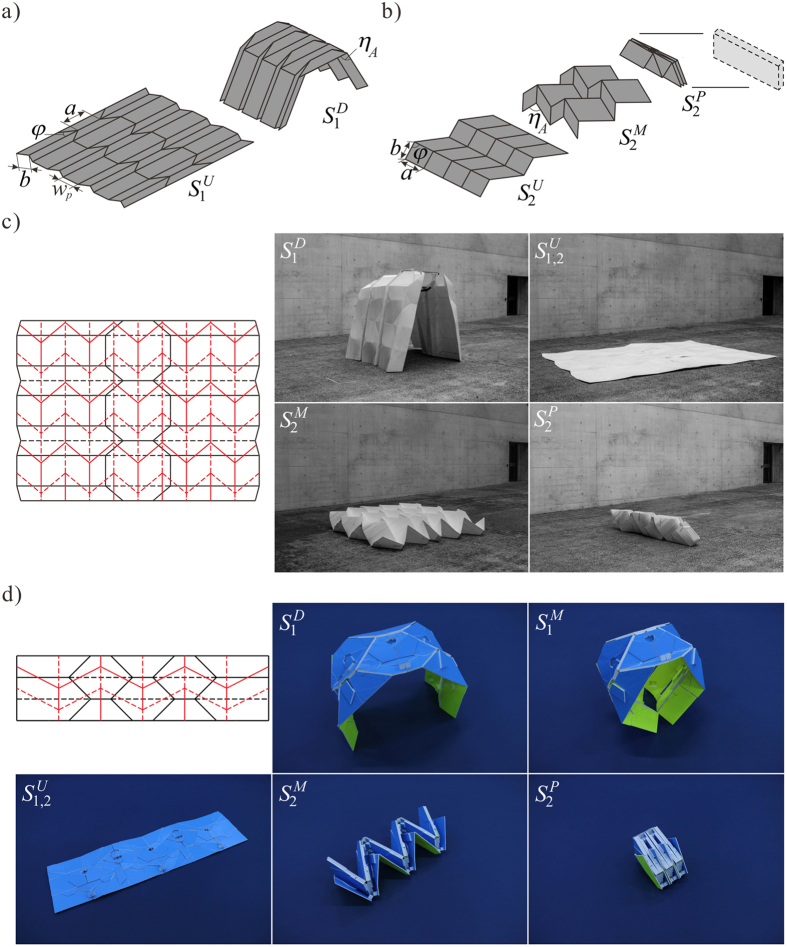
Foldable origami shelter with two independent states. (**a**) The crease pattern *S*_1_ and the folded shelter configuration. (**b**) The Miura-ori pattern *S*_2_ and its packaged configuration. (**c**) Patterns *S*_1_ in black and *S*_2_ in red are superimposed to form a pattern *S*_1,2_, with independent rigid motion between the shelter state *S*_1_^*D*^ and the package state *S*_2_^*P*^. (**d**) The thick-panel accordion shelter based on the superimposed Arc and Miura-ori patterns, with rigid motion between the two independent states.

A 1-DOF distributed frame accordion shelter^25^ is shown in Fig. 3a, with nearly flat-folded frame elements separated by spacer panels. In a deployed state *S*_1_^*D*^, the frame elements give the shelter a high structural stiffness but prevent the shelter from reaching a compact packaged state. This is a critical weakness for a deployable structure, which are almost always required to be packaged for transport. A second pattern, the 1-DOF Miura-ori shown in Fig. 3b, has an in-plane folding motion that can never self-intersect during folding and reaches a compact flat-folded state *S*_2_^*P*^ with a cuboid volume boundary.

Pattern superposition will allow the packaged configuration of *S*_2_ to resolve the packaging limitation of *S*_1_. One such combination *S*_1,2_ is shown in Fig. 3c which has necessary conditions for independent 1-DOF rigid-foldability, that is only cross-crease vertices are introduced during pattern superposition. Design flexibility is introduced in the superimposed pattern that warrants further inspection. *S*_1_ is specified with seven independent control parameters: sector angle* φ*, side lengths *a* and *b*, spacer plate width *w*_*p*_, folded edge angle *η*_*A*_, and number of tessellated units along *x* and *y* axes, *M* and *N*, respectively. *S*_2_ is similarly specified but without the spacer plate width parameter and so has six independent control parameters. Assuming *S*_1_ is the base pattern, design flexibility in specification of *S*_2_ is mostly preserved, with four of six parameters remaining free after the two tessellation unit length constraints are applied.

A full scale prototype of the crease pattern shown in Fig. 3c and SI Video 2 was constructed from a 2400 mm × 3600 mm × 3 mm extruded corrugated polypropylene sheet material. A 2 mm wide heated roller was used to compress the sheet along crease lines and the folded states were reached with manual folding by three people. As predicted by the kinematic analysis, the bifurcation in the unfolded state did not interrupt the folding motion of either state once the creases of a particular state were actuated. Good correspondence and independent foldability are seen between predicted and prototype folded forms at *S*_1_^*D*^ and *S*_2_^*P*^.

Transition to structural applications of origami geometry also necessitates the consideration of thick, rather than zero-thickness panels, with a comprehensive kinematic synthesis for rigid origami of thick panel presented in ref. 26. With a change from zero to non-zero sheet thickness, the physical polarity of crease lines is determined and so it is much easier to restore origami to a completely unfolded state and thus transition between states. A thick-panel multi-state accordion shelter was constructed as shown in Fig. 3d and SI Video 3, with the superimposed crease pattern generated from methods described above. Similar to the zero-thickness case, cross-crease vertices in the thick-panel model are able to maintain 1-DOF mobility for each embedded pattern and the prototype exhibits a smooth transition between both independent states.

### Sequent folding of superimposed patterns

Although previous superimposed patterns can realize transformation between embedded patterns, it can be seen that all shown state transformations were via the unfolded configuration, that is the independent mobility followed the process of ‘fold *S*_1_ – unfold – fold *S*_2_’. However there exist certain special superimposed patterns that can directly transform between states, that is a ‘fold *S*_1_ – fold *S*_2_’ transformation that skips the unfolded configuration.

**Figure 4:**
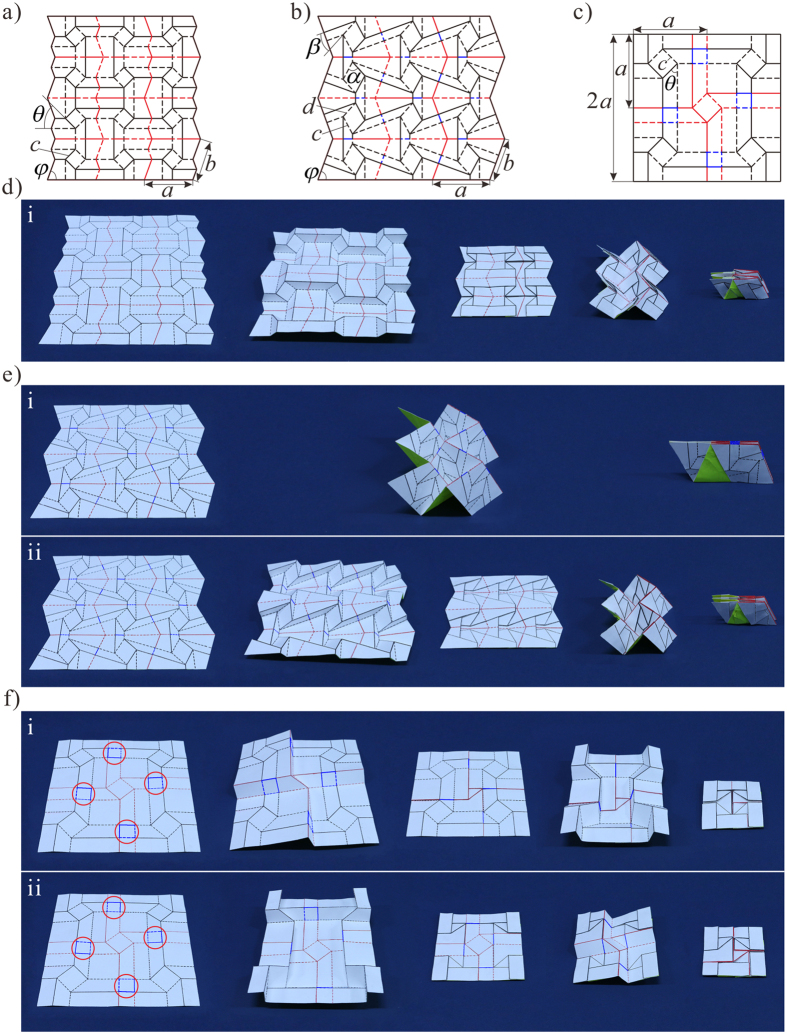
Sequent folding origami. (**a**) Superimposed pattern of square twist and Miura, with a modified boundary condition to permit between-state transformation. (**b**) Superimposed pattern of Miura-ori and double corrugated patterns. (**c**) Superimposed pattern of two square twist patterns. (**d**) Folding sequence of the pattern in (**a**). (**e**) Two folding sequences of the pattern in (**b**), with different final configurations. (**f**) Two folding sequences of the pattern in (**c**), with the same final configuration.

A superimposed pattern with square twist and Miura-ori embedded patterns can be obtained by following the superimposition process outlined previously. However, a modified process enables between-state transformation. A square twist pattern with sector angle *θ* and square length *c* is shown in Fig. 4a. It has a modified boundary condition with side lengths *a* and *b*, and sector angle *φ*, shown in black, such that when folded, its folded configuration corresponds exactly with a Miura-ori pattern boundary, as shown in Fig. 4d. A Miura-ori pattern with sector angle *φ*, shown in red, can thus be superimposed onto the folded square twist configuration and subsequently permit a second-stage of folding into the Miura-ori pattern. The between-state transformation enables a much more compact final package. The areal folding ratio of the first folding step being the folded state of square twist pattern is


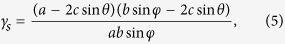


and that of Miura-ori pattern is


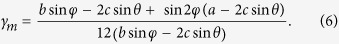


So the total areal folding ratio of superimposed pattern is


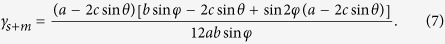


For the superimposed pattern in Fig. 4a, with *θ* =** 45°, *c* = 27.0 mm, *φ* = 70°, *a* = 92.2 mm, *b* = 82.8 mm, the areal folding ratio of two folding steps is *γ*_*s*_ = 0.30 and *γ*_*m*_ = 0.16, respectively. And the total areal folding ratio *γ*_*s*+*m*_ is 0.05. Referring back to the superimposed unfolded state, additional Miura-ori pattern crease lines are created due to the projection of the Miura-ori pattern ‘through’ the folded square twist panels. A prototype of the superimposed pattern and its full folding sequence are shown in Fig. 4d and SI Video 4.

Figure 4b shows a Miura-ori pattern, with sector angle* φ* and side lengths *a* and *b*, superimposed with a double corrugated unit, with sector angle *β*, parallelogram angle *α* and side lengths *c* and *d*. There are two permissible folding sequences, each of which ends with a fully folded Miura-ori, as shown in Fig. 4e and SI Video 5. The Miura-ori can be folded directly in a single step, or indirectly in two steps, with the first step being the folded state of the double corrugated pattern. For the indirect case, certain Miura-ori crease lines reverse their polarity in the second folding step, shown in blue, due to the overlap of crease lines in the double corrugated state. In order to ensure the overlap of crease lines in Miura-ori pattern after folding the double corrugated pattern, the crossed crease lines must be perpendicular to each other. The overlapped crease lines reduce the effective side length of the indirect Miura configuration and so the final packaged size is much smaller in the indirect case. The areal folding ratio of the direct case is


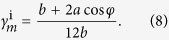


and the areal folding ratio of the indirect case is


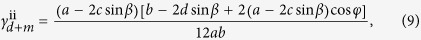


with


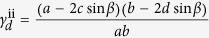


for the first double-corrugated folding step and


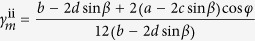


for the second Miura-ori folding step. For the superimposed pattern in Fig. 4b, with *φ* = 70°, *a* = 102.5 mm, *b* = 77.9 mm, *α* = 70°, *β* = 28°, *c* = 35.4 mm, *d* = 33.0 mm, the areal folding ratio of the first folding sequence is 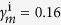 and the total areal folding ratio of the second folding sequence is 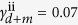.

Finally, Fig. 4c shows a superimposed pattern with two embedded square twist patterns. Each square twist pattern has identical sector angle *θ* and square length *c*, but with different side length, *a* for black and 2*a* for red. It again has two permissible folding sequences, each of which has two steps, either folding the red square-twist pattern first then the black one second, or vice versa, as shown in Fig. 4f and SI Video 6. Unlike the previous example though, both sequences fold into the same final square, despite the folded configuration of the first folding step of two sequences being different. The total areal folding ratio of the first folding sequence is


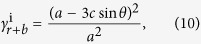


with


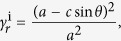



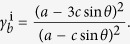


And the total areal folding ratio of the second folding sequence is


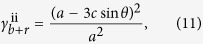


with


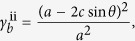



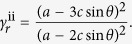


For the superimposed pattern in Fig. 4c, with *θ* =** 45°, *a* = 145.3 mm, *c* = 41.0 mm, the total areal folding ratio of the first folding sequence is 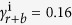, with 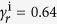 for the first folding step and 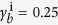 for the second. The total areal folding ratio of the second sequence is also 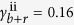, with 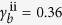 for the first folding step and 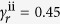 for the second. The folding sequence is able to be determined by controlling the order with which the blue crease lines fold, shown circled in Fig. 4f. For example, if the blue crease lines which are collinear with black lines remain collinear during the first folding step, then the black square twist pattern is folded first and the red twist pattern second, and vice versa.

## Discussion

The superimposition method presented in the paper combines two 1-DOF rigid-foldable origami patterns within one single sheet in such a way that only cross-crease vertices are generated. The particular kinematic properties of cross-crease vertices mean that the folding of one motion path suppresses the other, and vice versa. The superimposed pattern with cross-crease vertices thus has preserved 1-DOF mobility and is able to fold independently between each embedded state. Sequent folding between origami patterns is also shown to be possible and enables folding between different states without the requirement of transition through an unfolded state.

The ability to have two or more independent objects coexistent within a single sheet enables many new types of self-folding and origami-inspired engineering design possibilities. States with complementary functionality can be combined to give a device extended performance capabilities, as was seen in the example of a non-flat foldable shelter embedded with a compact packaged state. States with radically different functionality can also be combined to create multi-tool sheets capable of performing a broad range of functions. Finally, as the superposition method is purely enabled by the special cross-crease vertex, the principles of thick-panel and 1-DOF rigid origami design remain applicable and so the method can be applied across a range of material types and application domains.

## Additional Information

**How to cite this article**: Liu, X. *et al*. One-DOF Superimposed Rigid Origami with Multiple States. *Sci. Rep*. **6**, 36883; doi: 10.1038/srep36883 (2016).

**Publisher's note:** Springer Nature remains neutral with regard to jurisdictional claims in published maps and institutional affiliations.

## Supplementary Material

Supplementary Information

Supplementary Video S1

Supplementary Video S2

Supplementary Video S3

Supplementary Video S4

Supplementary Video S5

Supplementary Video S6
