# Supplementary material for: One-DOF Superimposed Rigid Origami with Multiple States
Source: Sci Rep. 2016 Nov 10;6:36883. doi: 10.1038/srep36883 (PMC5103280; doi:10.1038/srep36883)
Supplement: Supplementary Information [file srep36883-s1.docx]

**One-DOF Superimposed Rigid Origami with Multiple States**

Authors: Xiang Liu^1^, Joseph M. Gattas^2^, Yan Chen ^1,3^*

Address:

^1^ School of Mechanical Engineering, Tianjin University, Tianjin 300072, China

^2^ School of Civil Engineering, University of Queensland, St Lucia, QLD 4072, Australia

^3^ Key Laboratory of Mechanism Theory and Equipment Design of Ministry of Education, Tianjin University, Tianjin 300072, China

* Corresponding Author <yan_chen@tju.edu.cn>

**Supplementary information**

Videos:

1. Video 1: the physical model in Figure 1g;
2. Video 2: the physical model in Figure 3c;
3. Video 3: the physical model in Figure 3d;
4. Video 4: the physical model in Figure 4d;
5. Video 5: the physical model in Figure 4e; and
6. Video 6: the physical model in Figure 4f.
