# Supplementary figures and images for: One-DOF Superimposed Rigid Origami with Multiple States
Source: Sci Rep. 2016 Nov 10;6:36883. doi: 10.1038/srep36883 (PMC5103280; doi:10.1038/srep36883)

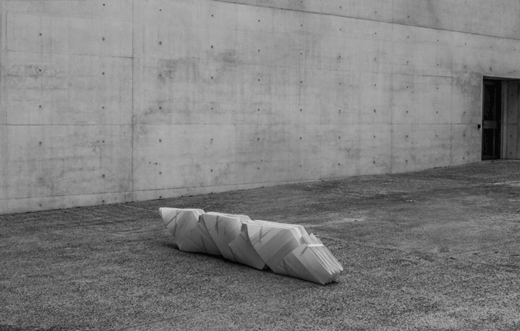

Supplement: Supplementary Video S1 [file srep36883-s2.gif]
